# Red Cross Conference on Venereal Diseases

**Published:** 1921-07-09

**Authors:** 


### Red Cross Conference on Venereal Diseases.

This Northern Europe Red Cross Conference, having considered the general measures for the combating of venereal diseases, which have been adopted by the participating countries, is unanimously of opinion, so far as the experience of the countries represented is concerned :—

1. That the provision, by responsible health authorities, of adequate facilities for diagnosis and treatment on lines which insure that the greatest possible number of infected persons is rendered non-infective is a measure of prime importance to the reduction of venereal diseases. The urgent necessity of commencing treatment at the earliest possible moment should be emphasised. It is suggested that the above facilities should be provided free of cost to the patient where they are otherwise unlikely to be utilised to the fullest extent.

2. That the questions of compulsory notification and of compulsory treatment, being dependent on the experience, resources and psychology of the people concerned in each country, must be decided by individual nations.

3. That instruction, theoretical and practical, in the recognition of venereal diseases, particularly in their earliest manifestations, and in their treatment, should form a part of the curriculum of every medical student, and that satisfaction of a test of proficiency in this subject should be a condition of every medical qualification.

4. That provision should be made, at suitable treatment centres, for such instruction of medical practitioners in the diagnosis and treatment of venereal diseases as will enable them to recognise these disabilities promptly and secure their adequate treatment.

5. That regulation and official toleration of professional prostitution have been found to be medically useless as a check on the spread of venereal diseases, and may even prove positively harmful, tending as they do to give official sanction to a vicious traffic.

6. That the provision of "hostels" and rescue homes for the temporary care of girls suffering from venereal disease is a valuable means of preventing the spread of venereal diseases.

7. That the provision of opportunities for wholesome entertainment and recreation is an important factor in reducing the temptation and exposure to venereal infections.

8. That enlightenment of the general public on lines which are best calculated to minimise exposure to infection and emphasise the necessity of thorough treatment, is an essential part of any scheme for the combating of venereal diseases. Instruction should particularly be addressed to parents and teachers in such a form as will enable them to give clear information on the reproduction of life and impress on adolescents the importance of individual responsibility to future generations. In the training of teachers special courses on these subjects should be provided.

9. That the Conference welcomes all efforts of the Red Cross Societies directed toward supplementing the efforts of official governmental agencies where the circumstances in the respective countries permit and indicate such activities, and in supporting the work of voluntary societies in the campaign against venereal diseases; and also welcomes the efforts of the League of Red Cross Societies in co-ordinating the activities of voluntary societies in this campaign throughout the world.

